# Coordination-Driven Controlled Assembly of Polyphenol-Metal Green Coating on Wood Micro-Grooved Surfaces: A Novel Approach to Stable Superhydrophobicity

**DOI:** 10.3390/polym9080347

**Published:** 2017-08-07

**Authors:** Kaili Wang, Zhong Wang, Youming Dong, Shifeng Zhang, Jianzhang Li

**Affiliations:** 1 Key Laboratory of Wood-Based Materials Science and Utilization, Beijing Forestry University, No. 35 Tsinghua East Road, Haidian District, Beijing 100083, China; wangkaili212@163.com (K.W.); wangzhong@bjfu.edu.cn (Z.W.); dongyouming@bjfu.edu.cn (Y.D.); 2 Beijing Key Laboratory of Wood Science and Engineering, Beijing Forestry University, No. 35 Tsinghua East Road, Haidian District, Beijing 100083, China

**Keywords:** polyphenol chemistry, multistep assembly, surface modification, superhydrophobicity, stability

## Abstract

A versatile, fast, and nature-inspired polyphenol chemistry surface modification was applied to prepare superhydrophobic surfaces with micro-grooved structures in this study. Tannic acid and iron ion (TA–Fe^III^) complexes were employed as a molecular building block for anchoring biomimetic coating onto the wood substrate with catalytically reducing formative Ag ions as the rough surface to ensure well-developed micro/nanostructure hierarchical roughness. TA–Fe^III^ complexes also acted as stable bridges between the substrate and hydrophobic groups. The thickness and architecture of TA–Fe^III^ complex coatings can be tailored by coordination-driven multistep assembly. The results indicated that the micro/nano hierarchical roughness structure was well-developed with increased coating times and increased deposition of reduced Ag nanoparticles, resulting in excellent superhydrophobic properties (e.g., water CA (contact angle) of about 156° and a rolling angle of about 4°). The superhydrophobic material exhibited outstanding stability and durability in harsh conditions, including strong acid/base or organic solvent, high-temperature water boiling, ultrasonic cleaning, and ultraviolet aging. A series of superhydrophobic models are proposed to clarify the effect of the micro/nano hierarchical structure on these superhydrophobic properties.

## 1. Introduction

Inspired by the charming performance of natural organisms, the design and fabrication of advanced functional materials has attracted a great deal of research attention in recent years [1]. The lotus leaf, which effectively self-cleans within its humid and muddy growing environment, is famous for its low-adhesive superhydrophobicity. Bionic, artificial superhydrophobic surfaces can be achieved by the combination of suitable hierarchical micro/nanostructures superimposed with low surface free energy compositions; these surfaces have notable potential in self-cleaning, anticorrosion, antifogging, anti-icing, drag reduction, and oil–water separation applications, among others [2,3].

Many artificial superhydrophobic surfaces have been designed and prepared by creating hierarchically-structured surfaces via the sol–gel technique, solvothermal methods, electrochemical processing, vapour deposition, etching, plasma treatment, template methods, and others [4,5,6,7,8,9,10]. There are notable disadvantages to these preparation methods, however, including tedious preparation processes or the necessity for harsh conditions, specialized equipment, or expensive or toxic reagents; existing preparation techniques even limit the types, sizes, and shapes of substrates available, which restricts their practical application [11].

The multistep assembly technique is a highly versatile way to assemble layered aggregates with tailored composition and architecture using very simple, inexpensive, and rapid procedures. It is particularly well-suited to coating deposition on non-flat surfaces with large areas [12]. Multistep assembly coatings with controllable thickness, architecture, and components would allow for micro/nano hierarchical structures with superhydrophobic properties. The most important part of multistep assembly is the effective and stable adhesion of substrate surfaces and assembly layers, as well as among the layers.

The natural adhesive ability of marine mussels has attracted a great deal of research attention in recent years. The adhesive ability of the mussel byssus is conferred by l-3,4-dihydroxyphenylalanine (DOPA) and the complexation between catechols in mussel foot protein-1 and iron has been identified as a key interaction [13,14]. Dopamine multifunctional surface modification has been developed as a versatile, green, and efficient approach to mimic the mussel surface. However, the expensive price, toxicity, and poor accessibility of dopamine restricts its wider application.

Plant polyphenols were proposed as an alternative to dopamine by Messersmith et al. [15] The plant phenols have abundant catechol/pyrogallol groups, and are very similar in structure and properties to dopamine. More importantly, they are superior in terms of their availability in nature, low cost, high adhesion rate, and non-toxicity [16].

Tannic acid (TA) is present in a variety of plants and fruits and is “generally recognized as safe” (GRAS) by the U.S. Food and Drug Administration [17]. TA is comprised of a central glucose core surrounded by covalently attached digalloyl ester groups containing luxuriant phenolic hydroxyl groups, which provide polydentate ligands for metal ions to form metal–polyphenol complexes [14,18]. Caruso et al. [17] found that TA can coordinate with Fe^III^ ions to form a highly stable TA–Fe^III^ complex coating which tightly adheres to the surfaces of nearly all manner of substrates via covalent and/or noncovalent bonding. Upon mixing TA and Fe^III^ in water at ambient temperature, the process is complete within minutes; further, the raw materials (TA and FeCl_3_) are both readily available and low in cost. More importantly, the thickness and architecture of TA–Fe^III^ complex coating can be tailored by coordination-driven multistep assembly, this allows the engineer to completely cover the original substrate surface and construct a new micro/nano hierarchical roughness surface. The free catechol/pyrogallol groups also have diverse secondary reactions, including catalytically reducing noble metal ions into nanoparticles or reacting with amino-containing or thiol-containing molecules through Michael addition or Schiff base reactions [19]. This process is suitable as a general approach to preparing micro/nano hierarchical surfaces with superhydrophobicity.

It is difficult to prepare superhydrophobic surfaces on micro-grooved structures [20,21]. Based on the model described by Cassie and Baxter [22], when water droplets are placed into micro-grooved structure surfaces, the air in the micro-grooves rapidly escapes in both directions, and the droplets fill the micro-grooves, exhibiting similar Wenzel’s states; this causes smaller water contact angles (CAs) and larger rolling angles (RAs) which harm the superhydrophobic properties. The proposed TA–Fe^III^ complex coating with controllable thickness and architecture by coordination-driven multistep assembly may be a favourable solution to these problems.

Wood, which possesses a unique anatomical structure and porosity, features a longitudinal surface characterized by concave lumen channel surfaces combined with cell wall ridges that form a micro-grooved structure [23]. Wood longitudinal surfaces were chosen as a naturally micro-grooved template for preparing superhydrophobic material in this study.

Most importantly, to the best of our knowledge, there has been no successful preparation of superhydrophobic surfaces with micro-grooved structures through depositing a thickness-controllable TA–Fe^III^ coating via a coordination-driven multistep assembly approach prior to this study. Therefore, in this study, a versatile, fast, and simple method was developed to prepare superhydrophobic surfaces with micro-grooved structures. 

## 2. Materials and Methods

### 2.1. Materials

Straight-grained sapwood portions of fast-growing poplar wood (*Populus tomentosa* Carr.) were manufactured into 3 × 20 × 20 mm^3^ (radial × tangential × longitudinal) blocks. TA (93% purity) and octadecanethiol (97% purity) were purchased from Tianjin Heowns Biochem Co., Ltd., (Tianjin, China) Iron(III) chloride hexahydrate (FeCl_3_·6H_2_O, analytical grade, 99% purity), anhydrous ethanol, toluene, acetone, hydrochloric acid, sodium hydroxide, n-hexane, and *N*,*N*-Dimethylformamide (DMF) were purchased from Beijing Chemical Works (Beijing, China).

### 2.2. Preparation of Superhydrophobic Surfaces on Wood Substrate

All wood samples were Soxhlet-extracted with toluene/ethanol/acetone (4:1:1 *v*/*v*/*v*) for 12 h to remove extractives, then put in an oven at 103 ± 2 °C until they reached a constant weight. TA (0.6 g) was dissolved in deionized water (300 mL) to form the solution (2.0 mg/mL). Wood samples were immersed into the TA solution, then FeCl_3_·6H_2_O (molar ratios of 3:1 between Fe^III^ and TA) aqueous solution was added to the TA aqueous solution. The solution was mixed by vigorous stirring for 30 s. The pH of the solution was adjusted to ca. pH 8 with 1 M NaOH solution, then the wood substrates were rinsed with water several times and dried in an oven at 60 °C. This completed the entire process of coating TA–Fe^III^ complexes on the wood surfaces.

We postulated that the multistep assembly protocol would enhance the micro/nano hierarchical roughness of the substrate. The coating process was repeated three times with the same process conditions as above. To further construct a well-developed micro/nano hierarchical structure, reduced Ag nanoparticles and aggregates were deposited onto the TA–Fe^III^ complex coating surfaces by immersing samples into AgNO_3_ solution (2.0 mg/mL) for 12 h, then washing them with water and drying them at 60 °C. All wood samples were soaked into an ethanolic solution of octadecanethiol (1:100 *v*/*v*) to react at 30 °C for 24 h, then washed with ethanol several times and dried at 60 °C. This yielded superhydrophobic wood with a micro/nano hierarchical surface. The samples prepared with different coating times and combined with Ag particles were labeled as 1st, 2nd, and 3rd/TA–Fe^III^/Wood, and 1st, 2nd, and 3rd/TA–Fe^III^/Ag/Wood, respectively.

### 2.3. Characterizations

#### 2.3.1. Micromorphology Analysis

Scanning electron microscopy (SEM) was performed on an FEI Quanta 650 scanning electron microscope (FEI, Hillsboro, OR, USA) with an acceleration voltage of 15 kV.

#### 2.3.2. Chemical Composition Analysis

X-ray photoelectron spectroscopy (XPS) was collected on a K-Alpha X-ray photoelectron spectrometer (Thermo Fisher Scientific Co., Ltd., Shanghai, China) with a monochromatic Al Kα source (1486.6 eV).

#### 2.3.3. Characterization of Superhydrophobic Properties

Contact angles (CAs) and Rolling angle (RAs) were measured on a Dataphysics OCA 20 (Dataphysics, Filderstadt, Germany) instrument as reported in the literature [24,25]. The CAs were measured at six different points of each sample with ellipse fitting modes. The RAs were determined by releasing a water droplet (about 10 μL) onto a wood surface, then the sample platform was tilted until the water droplet rolled off the surface; the critical angles of inclination were recorded as RAs.

#### 2.3.4. Characterization of Superhydrophobic Stabilities

The wood samples were immersed into HCl solution (pH = 2), NaOH solution (pH = 12), and various organic solvents for 24 h and the CAs were measured to demonstrate the superhydrophobic surfaces’ chemical durability. The resistance to ultrasonic cleaning of the superhydrophobic wood was tested by submerging the samples in distilled water six times for a total of 1 h under ultrasonication (40 kHz frequency, 100 W). The samples were collected at certain intervals, and dried in an oven at 100 °C for 3 h followed by CA measurements. We next placed the superhydrophobic wood samples in an ultraviolet aging test chamber (Beijing Beifang Lihui Instrument Equipment. Co., Ltd., Beijing, China) for a week (power: 40 W; radiation wavelength: 340 nm) and assessed their resistance to the treatment.

## 3. Results and Discussion

### 3.1. Preparation Process and Reaction Mechanism

**Figure 1:**
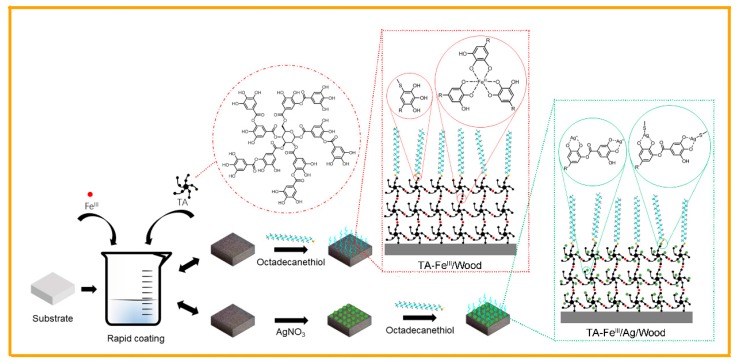
Preparation procedure and reaction mechanism for the superhydrophobic surface*.*

Figure 1 shows the bionic superhydrophobic surface preparation process. TA provides polydentate ligands for Fe^III^ ion coordination, forming the TA–Fe^III^ complex coating which adheres on the substrate surface via covalent and/or non-covalent bonding by utilizing hydroxyl groups from catechol and/or galloyl. To ensure the complete coverage of the original substrate surface and obtain a well-developed surface morphology with suitable micro/nano hierarchical roughness, the wood samples were dipped into modification solutions several times and the TA–Fe^III^ complex coatings were gradually thickened with the increased times of the assembly process. The TA–Fe^III^ complex coating can reduce the Ag ion into Ag nanoparticles, which further enhances the micro/nano hierarchical roughness. Meanwhile, TA and Ag particles react with thiol-terminated molecules through the free catechol/pyrogallol-thiol reaction and metal-thiol coordination, respectively, grafting the hydrophobic long-chain onto the TA–Fe^III^ coating surface to achieve the superhydrophobic modification of the substrate.

Wang et al. prepared highly hydrophobic wood surfaces via the sol–gel method, where the water CA on longitudinal surfaces was only 140° due to the inability to fill the wood micro-grooved structure with the hydrophobic micro/nano coating [20]. The hydrothermal approach is also a traditional technique for fabricating superhydrophobic surfaces, but this process is pretty complicated, and not suitable for large-area fabrication. It also damages wood substrate structures and components because of the harsh environment it necessitates [26,27]. The process we propose allows for nature-inspired polyphenol chemistry surface modification in a versatile, simple, and green manner that does not damage the intrinsic structures or components of the wood substrates. The thickness and architecture of the TA–Fe^III^ complex coating can also be fine-tuned via coordination-driven multistep assembly to fully cover the original substrate surface and form a new micro/nano hierarchical roughness structure. This mitigates the poor superhydrophobic performance of uncoated hydrophilic substrates exposed on the surface. The as-prepared superhydrophobic surfaces in this study also exhibited excellent stability in harsh conditions due to the strong interfacial interaction.

### 3.2. Micromorphology and Chemical Composition Analysis

**Figure 2:**
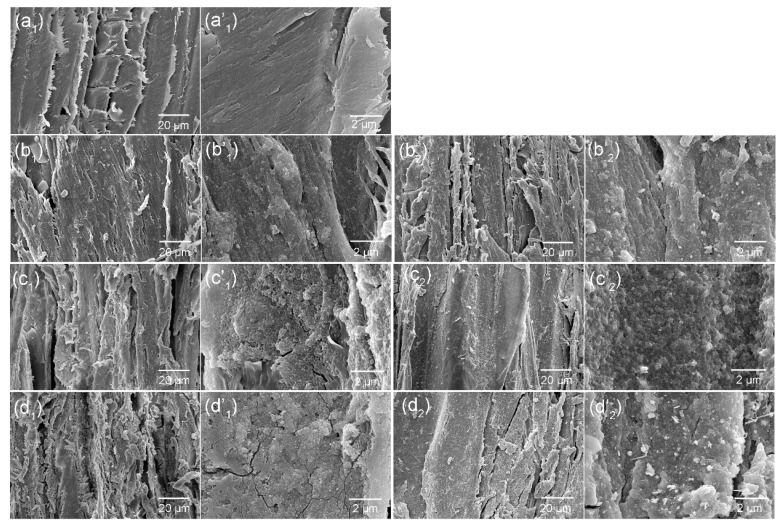
SEM (Scanning electron microscopy) observations of tangential sections of (**a_1_**) control wood, (**b_1_**–**d_1_**) 1st, 2nd, and 3rd /TA–Fe^III^/Wood, and (**b_2_**–**d_2_**) 1st, 2nd, and 3rd /TA–Fe^III^/Ag/Wood at different magnifications.

Figure 2 shows the surface morphologies of the control wood, TA–Fe^III^/Wood, and TA–Fe^III^/Ag/Wood samples at different magnifications. The avulsed lamellar structure of the cell walls with smooth lumen surfaces are shown in control wood, forming an alternating “valley” and “ridge” roughness structure at microscale level (Figure 2a). After the wood samples were rapidly coated with TA–Fe^III^ complexes, a thin and rough layer was observed on the lumen surface; the surface had much rougher structure as the coating time increased (Figure 2(b_1_–d_1_)). The excellent adhesion capacity and reactivity of the TA–Fe^III^ complexes facilitated a micro/nano hierarchical structure after immobilizing Ag nanoparticles and aggregating onto the wood surface because of the reductive ability of the catechol/pyrogallol groups in the TA. The deposited Ag nanoparticles and aggregates were clearly observed on the TA–Fe^III^ complex-coated surface (Figure 2(b_2_–d_2_)). A similar approach was previously reported for reducing Ag ion to Ag nanoparticles by TA reduction to secure excellent adhesion and stability [28]. In this study, TA–Fe^III^ complex coating and Ag nanoparticles synergistically served as building blocks to create micro/nano multiscale hierarchical structures; a superhydrophobic surface was successfully prepared after grafting hydrophobic groups. 

**Figure 3:**
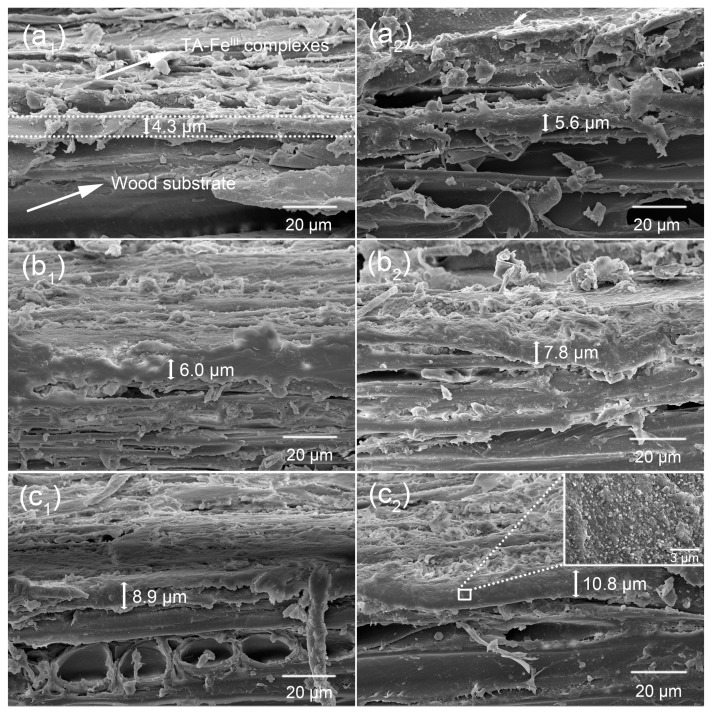
SEM observations of superhydrophobic coating thickness (top-front views). (**a_1_**–**c_1_**) TA–Fe^III^ complexes coated tangential-section (top) and original radial-section (front) in 1st, 2nd, and 3rd/TA–Fe^III^/Wood, (**a_2_**–**c_2_**) TA–Fe^III^/Ag coated tangential-section (top) and original radial-section (front) in 1st, 2nd, and 3rd/TA–Fe^III^/Ag/Wood.

Figure 3 shows the process of thickening of the TA–Fe^III^ layer-based superhydrophobic coating with multistep assembly. As shown in Figure 3(a_1_), the TA–Fe^III^ complex hydrophobic layer was about 4.3 μm thick on the wood substrate surface. Some “ridge” areas in the substrate were uncoated, likely resulting in superhydrophobicity with higher adhesive force. The coating thickness gradually increased about from 4.3 μm to 8.9 μm as the multistep assembly process continued (Figure 3(a_1_–c_1_)). When Ag nanoparticles and aggregates were deposited on the as-formed TA–Fe^III^ complex layers, the layer thickness increased about from 5.6 μm to 10.8 μm as coating time increased (Figure 3(a_2_–c_2_)). We observed complete coverage of the original wood surfaces (and enhanced superhydrophobic performance) with gradually thicker complex coatings. The inset image in Figure 3(c_2_) shows the longitudinal section of the superhydrophobic coating, which clearly displays a micro/nano hierarchical roughness structure.

In previous studies, the thicknesses of coordination-driven TA–Fe^III^ complex coating on various particles, capsules, and films were limited to several nanometers [14,17]. Our coating thickness reached the micrometer scale according to our SEM observations. This is because wood as a biopolymer composite possesses an interconnected network of micro-pores due to its inherent structure, resulting in a high level of hygroscopicity. When the wood samples were rapidly immersed into the TA solutions, the solutions partially penetrate into the wood substrate; upon adding the Fe^III^ and NaOH solution, the TA–Fe^III^ complexes formed not only on substrate surface but also on the “penetration” layer, which conformed to the previous literature [29,30]. The superhydrophobic coating we created is effectively a hybrid layer including the wood substrate, TA–Fe^III^ complexes, reduced Ag nanoparticles, and grafted long-chain groups. Denser and thicker superhydrophobic coatings were formed with increased coating times and the immobilization of reduced Ag nanoparticles, allowing us to successfully and easily establish micro/nano hierarchical roughness structures.

**Figure 4:**
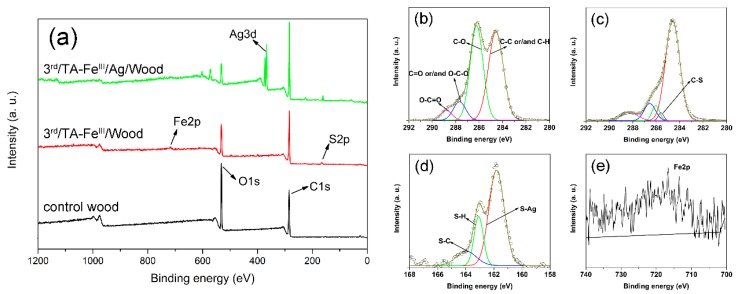
XPS (X-ray photoelectron spectroscopy) wide-scan (**a**), C1s core-level spectra of (**b**) control wood and (**c**) 3rd/TA–Fe^III^/Wood, S2p core-level spectra of (**d**) 3rd/TA–Fe^III^/Ag/Wood, and Fe2p core-level spectra of (**e**) 3rd/TA–Fe^III^/Ag/Wood.

The surface chemical component for the treated wood surface were analyzed via XPS as shown in Figure 4. The control wood only showed C and O signals (Figure 4a). As expected, characteristic Fe and S peaks appeared in the spectrum for 3rd/TA–Fe^III^/Wood and 3rd/TA–Fe^III^/Ag/Wood samples, indicating that TA–Fe^III^ complexes were successfully coated onto the wood surface and that long-chain hydrophobic alkyl was grafted onto TA–Fe^III^ complex surfaces after octadecanethiol treatment. The Ag3d signal was observed in 3rd/TA–Fe^III^/Ag/Wood samples, indicating that Ag particles formed on the TA–Fe^III^ complexes surface.

Figure 4b,c shows the C1s core-level spectra of control wood and 3rd/TA–Fe^III^/Wood samples. According to the classification of carbon atoms in wooden materials, the C1s peak was curve-fitted with four peak components with binding energies at 284.6 eV for C–C and/or C–H species, at 286.2 eV for C–O species, at 287.6 eV for C=O and/or O–C–O species, and at 288.8 eV for O–C=O species [31]. After TA–Fe^III^ complex coating and octadecanethiol treatment, the C1s core-level spectra of 3rd/TA–Fe^III^/Wood changed to five peak components; the new peak at 285.7 eV was probably attributable to the C–S species [32,33].

The Fe2p signal in Figure 4e shows the formation of TA–Fe^III^ complexes, and Figure 4d shows the S2p core-level spectra of 3rd/TA–Fe^III^/Ag/Wood which we curve-fitted with three peak components corresponding to S–Ag species at 161.8 eV, S–H species at 163.0 eV, and S–C species at 163.9 eV [34,35]. These observations indicate that the TA–Fe^III^ complex coating catalytically reduced the Ag ions into Ag nanoparticles and formed a Ag–S bond. C–S bonds between the TA–Fe^III^ complex layers and sulfur atoms allowed free catechol/pyrogallol groups in TA to react with –SH groups. These results indicated that the hydrophobic groups were successfully grafted onto the TA–Fe^III^/Ag complex surfaces.

### 3.3. Superhydrophobic Properties

**Figure 5:**
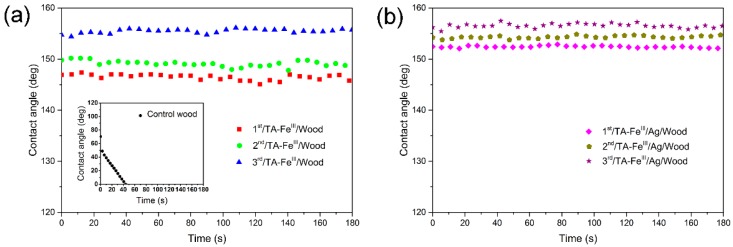
Contact angle as a function of time for (**a**) control wood, 1st, 2nd, and 3rd/TA–Fe^III^/Wood, and (**b**) 1st, 2nd, and 3rd/TA–Fe^III^/Ag/Wood.

Figure 5 shows the changes in CA on the wood longitudinal surface over time. The CAs of the control wood decreased rapidly within a short amount of time. The treated wood samples showed no remarkable change in CA, all remained above 146° by 180 s, indicating excellent hydrophobic properties. The CAs of TA–Fe^III^/Wood samples increased from about 146° to 154° with increased coating times, but increased from about 152° to 156° for TA–Fe^III^/Ag/Wood samples. The CAs of TA–Fe^III^/Ag/Wood were higher than the TA–Fe^III^/Wood samples under the same coating times.

**Figure 6:**
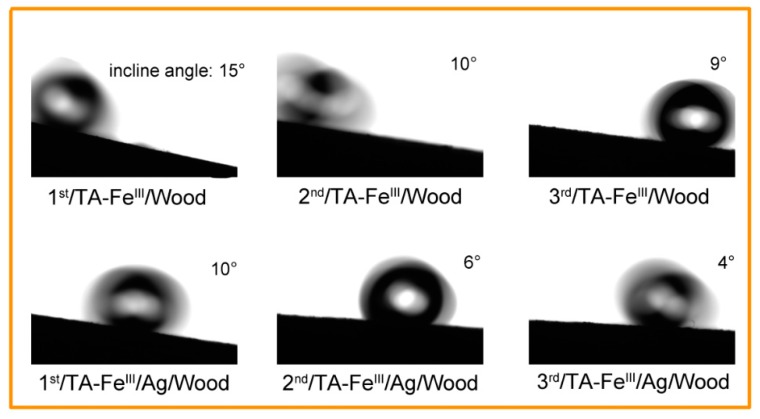
Water droplet profiles on inclined superhydrophobic wood sample surface.

The profile of water droplets as they rolled down the inclined sample surface is shown in Figure 6. The spherical water droplets rolled down the 1st/TA–Fe^III^/Wood at a 15° incline angle. With increased coating times, the angle decreased to 9° for 3rd/TA–Fe^III^/Wood and from 10° to 4° for TA–Fe^III^/Ag/Wood samples. These results demonstrate that a more suitable superhydrophobic, hierarchical roughness surface was constructed with increased TA–Fe^III^ complex coating times together with the deposition of as-reduced Ag particles and aggregates.

**Figure 7:**
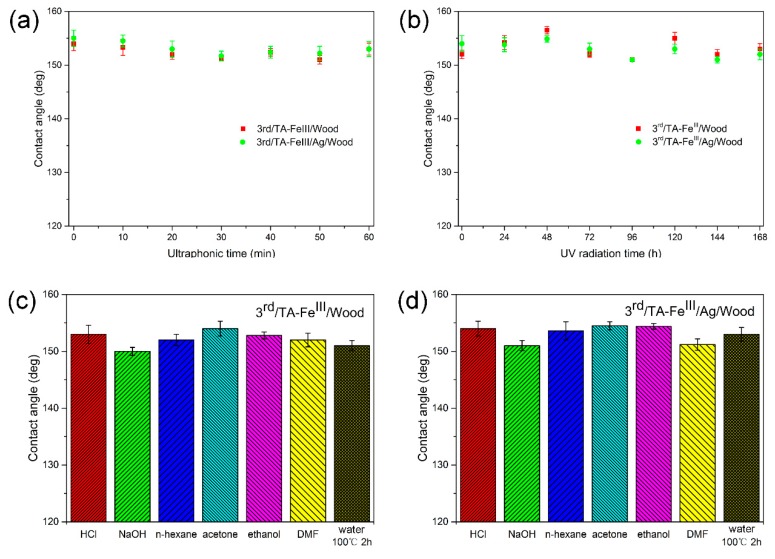
Stability and durability characterizations. (**a**) CAs (Contact angles) as a function of time after ultrasonic cleaning in water; (**b**) CAs as a function of UV radiation exposure time; (**c**,**d**) CAs for 3rd/TA–Fe^III^/Wood and 3rd/TA–Fe^III^/Ag/Wood samples soaked in different chemical solutions.

The superhydrophobic performance of treated wood samples after ultrasonic washing (40 kHz, 100 W) for 60 min was evaluated. The CAs of the 3rd/TA–Fe^III^/Wood and 3rd/TA–Fe^III^/Ag/Wood samples all remained larger than 150° after ultrasonic cleaning (Figure 7a), indicating the outstanding adhesive properties of our TA–Fe^III^ complex coating. Figure 7b shows the stability of the samples’ superhydrophobic properties after long-term UV radiation: The CAs of the treated wood surface remained above 150° after 168 h radiation, indicating outstanding UV resistance. Figure 7c,d shows the CAs of the 3rd/TA–Fe^III^/Wood and 3rd/TA–Fe^III^/Ag/Wood samples after dipping into various chemical reagents (HCl, pH = 2, NaOH, pH = 12, *n*-hexane, acetone, ethanol, and DMF) for 24 h, and 100 °C boiled water for 2 h. The CAs values were all above 150°. Taken together, these results demonstrate that the as-prepared bionic superhydrophobic surfaces exhibited excellent stability and durability in harsh conditions. 

Different methods have been developed to prepare superhydrophobic wood surfaces, but the poor environmental stability restricted its practical application. In some studies, the CAs on the treated superhydrophobic wood surfaces gradually decreased with immersion into various harsh conditions, including ultrasonic cleaning, strong acid/base or organic solvents, etc. [36,37,38]. In this study, the coordination-driven multistep assembly approach was able to form a stable superhydrophobic coating on wood surfaces, which possessed outstanding performance against various harsh conditions. 

### 3.4. Superhydrophobic Model Establishment

For a liquid droplet on a rough solid surface, superhydrophobic states can be described by three wetting models: The Wenzel model, the Cassie model, and a combination of the two, representing the transition from one to the other [2]. In Wenzel’s model, the droplet maintains contact with the surface and penetrates the asperities while the solid–liquid contact area is increased, causing stronger adhesion. Cassie’s model emphasizes that the droplet is suspended on the asperities, where trapping air in voids on the substrate surface tends to reduce the solid–liquid contact area, causing droplets to readily roll off the surface with low adhesion. A transitional model exists when the water droplets contact most samples [39].

We used the Cassie–Baxter equation to further elucidate the superhydrophobic properties of the treated surfaces. The equation is generally applicable to hierarchical or heterogeneous substrates [40].

(1) $$\cos\theta_{c}=f\left(\cos\theta +1\right)-1$$

where ƒ is the apparent area fraction of the solid surface contacting the liquid; the fraction of trapped air in contact with liquid at the surface is 1−ƒ; and θ and θ_c_ represent the CAs on smooth and rough surfaces, respectively. In this equation, θ is a constant value for a certain material.

**Figure 8:**
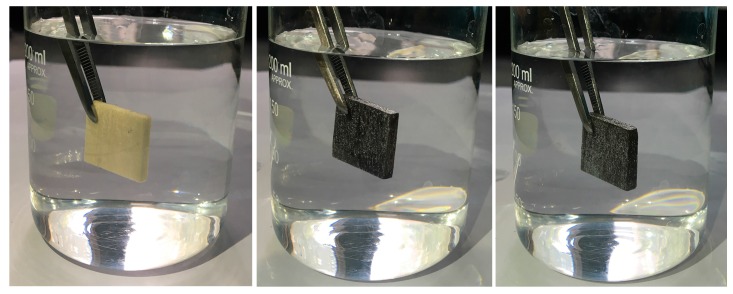
Mirror-like phenomena, from the left to right are control wood, 3rd/TA–Fe^III^/Wood, and 3rd/TA–Fe^III^/Ag/Wood samples, respectively.

The CA of water on the long-chain, alkyl-coated, smooth surface is 94.8° [41]. The trapped air fractions contacting droplets on the as-prepared surfaces can be calculated based on this value. The trapped air fraction values in contact with water on 1st, 2nd, and 3rd/TA–Fe^III^/Wood samples were 0.82, 0.85, and 0.90; on 1st, 2nd, and on 3rd/TA–Fe^III^/Ag/Wood samples they were 0.88, 0.89, and 0.91, respectively. These results indicate that air occupied about 82%–91% of the area contacting a water droplet on the as-prepared rough surface. The 3rd/TA–Fe^III^/Wood and 3rd/TA–Fe^III^/Ag/Wood sample surfaces both exhibited mirror-like phenomena when observed at an oblique angle under water (Figure 8), this is a signature of trapped air and the formation of composite solid–liquid–air interfaces. A higher air fraction contributes to a larger CA and a smaller RA [11]. The variations in CAs and RAs are shown in Figure 5 and Figure 6.

**Figure 9:**
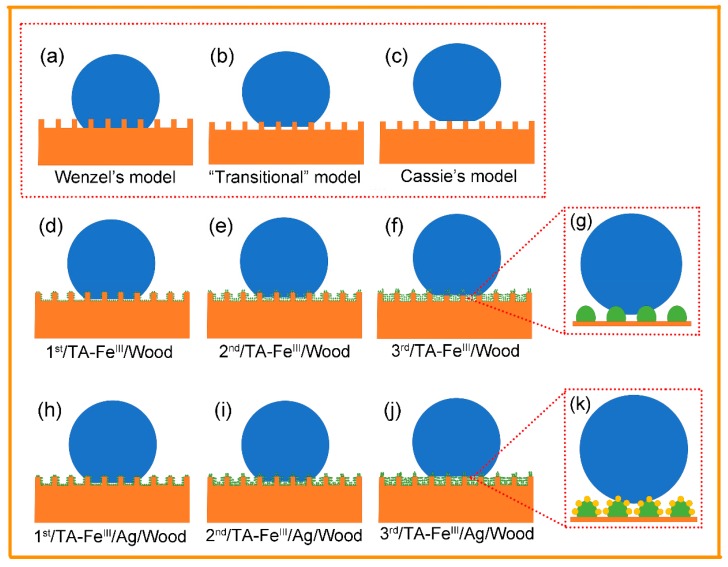
Superhydrophobic models. (**a**) Wenzel’s model; (**b**) “Transitional” model; (**c**) Cassie’s model; (**d**–**f**) proposed the 1st, 2nd, and 3rd/TA–Fe^III^/Wood samples superhydrophobic model; (**g**) proposed the surface morphology of TA–Fe^III^/Wood; (**h**–**j**) proposed the 1st, 2nd, and 3rd/TA–Fe^III^/Ag/Wood samples superhydrophobic model; and (**k**) proposed the surface morphology of TA–Fe^III^/Ag/Wood.

To clarify the as-prepared micro/nano hierarchical structure of substrate surfaces with increased coating times and immobilization of reduced Ag nanoparticles and aggregates, as well as the relation of these factors to superhydrophobic properties, we built a series of superhydrophobic models (Figure 9d–k). TA can coordinate with Fe^III^ ions in a short time to form a TA–Fe^III^ complex coating which tightly adheres to the substrate via covalent and/or noncovalent bonding. In the 1st/TA–Fe^III^/Wood sample (Figure 9d), the surface was coated onto a very thin layer. Although the hydrophobic properties improved dramatically after hydrophobic treatment, the CA and RA values did not break the superhydrophobic boundary, likely due to the presence of uncoated hydrophilic substrates exposed on the surface. With increased coating times, the complex particles were more densely spread out and the coating thickness gradually increased, which filled the micro-groove structures and established a better rough surface. Accordingly, when the water droplet was placed on the 3rd/TA–Fe^III^/Wood surface (Figure 9f), the as-formed nanoparticles and aggregates trapped air in the micro-grooves, reducing the solid–liquid contact area; this created an approximate Cassie’s state resulting in the larger CAs and smaller RAs. After the immobilization of the reduced Ag nanoparticles and aggregates on the TA–Fe^III^ complex coating surface, the formed micro/nano structure model was established as shown in Figure 9k. Compared to the TA–Fe^III^/Wood (Figure 9g), the TA–Fe^III^/Ag/Wood exhibited higher CAs and lower RAs under the same coating times. This is because a well-developed micro/nano hierarchical structure formed after Ag nanoparticles were deposited on the TA–Fe^III^ coating, which trapped more air and further decreased the solid–liquid contact area while improving the superhydrophobicity. 

These results also can be explained by the “Kao diagram”, which is a powerful tool to synthetically analyse the combined hydrophobicity/hydrophilicity of surfaces with homo/heterogeneity, and correlate physicochemistry and wettability of the modified material surfaces. The superhydrophobicity can be tuned by introducing changes in micro/nanoarchitecture of the surface or by surface chemical modification, which synergistically affected the superhydrophobic performance and surface energy [42,43,44,45,46]. Therefore, the effect of the well-developed micro/nanoarchitecture of surfaces on superhydrophobic properties can be explained by the “Kao diagram”. The well-developed micro/nanostructure domains were introduced onto the surface, which can be classified as the Cassie–Baxter region. In this zone, superhydrophobic surfaces possessing well-developed surface roughness were represented.

The gradually developed micro/nano hierarchical roughness structure with increased coating times and deposition of reduced Ag nanoparticles contributed to improved superhydrophobicity, higher CAs, lower RAs, decreased surface free energy, and a decreased solid–liquid contact area. At a certain number of coating times, the prepared 5th/TA–Fe^III^/Wood and 5th/TA–Fe^III^/Ag/Wood samples showed similar superhydrophobicity to the 3rd/TA–Fe^III^/Wood and 3rd/TA–Fe^III^/Ag/Wood samples in terms of CA, RA, and surface free energy. This suggests that the coating thickness was sufficient to fully cover the micro-grooved structure surfaces; the roughness morphologies did not change even after further assembly but instead the surface micro/nano layer simply grew thicker.

## 4. Conclusions

In this study, a versatile, fast, green, and low-cost surface modification method with controllable coating thickness was developed based on natural polyphenol chemistry to prepare superhydrophobic surfaces with micro-grooved structures. A micro/nano hierarchical roughness structure was fabricated first by the rapid coordination-driven multistep assembly of a TA–Fe^III^ complex coating on the substrate surface, forming a combination of bio-anchored Ag nanoparticles and aggregates. Secondly, thiol-containing hydrophobic groups were grafted onto the obtained micro/nano roughness surfaces through Michael addition or Schiff base reaction to achieve superhydrophobicity.

This method has several advantages.

- The TA–Fe^III^ complexes employed as a molecular building block for anchoring biomimetic coatings onto substrates also act as stable bridges between the substrate and hydrophobic groups. They also affect the reducibility of Ag nanoparticles, which endow the TA–Fe^III^ complex coating with micro/nanostructure hierarchical roughness.
- The thickness and architecture of the TA–Fe^III^ complex coating can be tailored by coordination-driven multistep assembly. The coating can fully cover the original substrate surface, even quite rugged surfaces with a large area, preventing any damage to the superhydrophobic performance due to uncoated hydrophilic substrates exposed on the surface.
- The whole procedure can be conducted under mild, eco-friendly conditions without destroying the substrate’s intrinsic structures or components.
- The TA–Fe^III^ coordination process is fast, simple, versatile, and the raw materials are readily available and low in cost.
- The bionic-prepared superhydrophobic surfaces showed excellent stability and durability in harsh conditions.

We also built a series of superhydrophobic models showing that increased coating times and the deposition of reduced Ag nanoparticles and aggregates gradually thickened the superhydrophobic coating and roughness structure. This resulted in improved superhydrophobicity, larger CAs, smaller RAs, decreased surface free energy, and decreased solid–liquid contact area. The concept of utilizing nature-inspired polyphenol chemistry to prepare superhydrophobic surfaces conforms well to the principles of green chemistry that are now integral to sustainable engineering. The method proposed here may allow for a wide range of potential applications in biomimetic materials.
